# Light Pollution Modifies the Expression of Daily Rhythms and Behavior Patterns in a Nocturnal Primate

**DOI:** 10.1371/journal.pone.0079250

**Published:** 2013-11-13

**Authors:** Thomas Le Tallec, Martine Perret, Marc Théry

**Affiliations:** Mécanismes Adaptatifs et Evolution, UMR 7179, Centre National de la Recherche Scientifique, Muséum National d'Histoire Naturelle, Brunoy, France; Pennsylvania State University, United States of America

## Abstract

Among anthropogenic pressures, light pollution altering light/dark cycles and changing the nocturnal component of the environment constitutes a threat for biodiversity. Light pollution is widely spread across the world and continuously growing. However, despite the efforts realized to describe and understand the effects of artificial lighting on fauna, few studies have documented its consequences on biological rhythms, behavioral and physiological functions in nocturnal mammals. To determine the impacts of light pollution on nocturnal mammals an experimental study was conducted on a nocturnal primate, the grey mouse lemur *Microcebus murinus*. Male mouse lemurs (N = 8) were exposed 14 nights to moonlight treatment and then exposed 14 nights to light pollution treatment. For both treatments, chronobiological parameters related to locomotor activity and core temperature were recorded using telemetric transmitters. In addition, at the end of each treatment, the 14^th^ night, nocturnal and feeding behaviors were explored using an infrared camera. Finally, throughout the study, body mass and daily caloric food intake were recorded. For the first time in a nocturnal primate, light pollution was demonstrated to modify daily rhythms of locomotor activity and core temperature especially through phase delays and increases in core temperature. Moreover, nocturnal activity and feeding behaviors patterns were modified negatively. This study suggests that light pollution induces daily desynchronization of biological rhythms and could lead to seasonal desynchronization with potential deleterious consequences for animals in terms of adaptation and anticipation of environmental changes.

## Introduction

Biodiversity faces a global decline. Most indicators of biodiversity show decreasing trends whereas most indicators of pressures on biodiversity show increasing trends [1]. Urbanization, suburbanization and associated anthropogenic activities exercise a strong constraint on living organisms and ecosystem functioning. These pressures can be direct (*e.g.* habitat modification and fragmentation) or indirect (*e.g.* altered temperature; air, noise and light pollution) [2]. Among anthropogenic pressures, light pollution exerts a strong force of selection on biodiversity [3]–[6]. Described for the first time in the 1880s [7], when Thomas Edison marketed the first electric bulbs, the effects of light pollution on living organisms and ecosystems were nevertheless long underestimated. A century later, Smith defined light pollution when the artificial sky brightness is greater than 10 per cent of the natural night sky brightness above 45° of elevation [8]. According this definition, Cinzano *et al.* [9] characterized the extent of light pollution across the world showing that light pollution impacted 85.3% of the surface area in the European Union, 61.8% in the United States (excluding Alaska and Hawaii) and 18.7% of emerged lands at the global scale. In addition, the increase of light pollution was estimated around 10% per year in the European countries [9], [10]. Referring to these studies, it could be estimated that the European countries are saturated by light pollution today. More recently, the increase of light pollution was estimated near 6% per year (range: 0–20%) across the world [3]. However, altering daily and seasonal cycles of natural light, light pollution impacts living organisms and ecosystems. Since several decades, it has been shown that light pollution affects most taxa (vertebrates, invertebrates and plants [11]). In addition, 28% of vertebrates and 64.4% of invertebrates live exclusively or partially at night, *i.e.* many species susceptible to be disturbed [4]. Most studies of light pollution have been conducted on nocturnal insects, reptiles (mainly sea turtles) and birds. Attraction of nocturnal insects to streetlights [12], [13], alteration of reproductive behavior in sea turtles near illuminated beaches [14] and misorientation/disorientation of birds near urban sky glow are the best documented impacts of light pollution [15], [16]. In contrast, the effects of light pollution on mammals have been largely underinvestigated [17].

In mammals, as in most animals, natural light and its cycles are fundamental for spatial and temporal representation of the environment. Natural light supplies crucial visual information and also non-visual information required to synchronize the internal biological clock with the geophysical cycles on Earth. In terms of chronobiology, *i.e.* in terms of temporal organization of living organisms, this synchronization allows to adapt to the environment [18]–[20]. Under light pollution, could the light information perceived by mammals be modified and the daytime artificially lengthened leading to disturbed behaviors, biological rhythms and physiological functions as suggested by Shuboni *et al.* [21]? Most studies on light pollution effects in mammals have been conducted in rodents and bats. They stressed a decrease of locomotor activity and a delay of daily emergence activity [22]–[26], a reduction of foraging and modifications of feeding behavior [25], [27]–[29] and a negative impact on juvenile growth, body mass and immune function [23], [30] after exposure to light pollution. However, to our knowledge, except recent works as those conducted by Rotics *et al.* [26], [31], few experimental studies explore in detail the impact of light pollution on biological rhythms in mammals, especially on daily rhythms of locomotor activity and core temperature, both parameters conventionally used in chronobiology.

To better understand the consequences of urban light pollution on mammals, we conducted an experimental study on a nocturnal primate, the grey mouse lemur (*Microcebus murinus*). The grey mouse lemur, a Malagasy prosimian species representative of the common ancestor of primates [32], is a convenient model to study the impact of light pollution because of the strong dependence of its behaviors, biological rhythms and physiological functions on the photoperiod in the wild and in captivity. The annual rhythm of the mouse lemur is characterized by an active sexual state in long-days photoperiod (>12 hours light), an inactive sexual state and an increase in adipose mass in short-days photoperiod (<12 hours light) [33]. The daily rhythm is characterized by an important locomotor activity with euthermy at night and low locomotor activity with a phase of hypothermia during the day [34]. In this study, we used a quantitative approach based on chronobiological tools to investigate the impact of light pollution on parameters related to daily rhythms of locomotor activity and core temperature. Jointly we observed nocturnal and feeding behaviors in relation to body mass. Considering the dependence of daily rhythms on photoperiod and considering that light pollution could modify the light information perceived by animals and artificially lengthen daytime, we predicted a phase delay in daily rhythms of locomotor activity and core temperature and a negative effect on nocturnal and feeding behaviors during exposure to light pollution.

## Materials and Methods

### Ethics statement

All experiments were performed in the laboratory breeding-colony of Brunoy (UMR 7179 CNRS/MNHN, France; agreement n° E91-114-1 from the Direction Départementale de la Protection des Populations de l'Essonne) under the authorization n° Ce5/2011/067 from the Charles Darwin Ethics Committee in Animal Experiment and the Internal Review Board of the UMR 7179. All experiments were done under personal license to T. Le Tallec (authorization n° A91–621 from the Direction Départementale de la Protection des Populations de l'Essonne). Surgery was performed under veterinary supervision (DVM F. Aujard n° 17–145) and all efforts were made to minimize nociception. Human endpoints were determined as follows: significant loss of body mass (body mass <60 g); inactivity associated with a state of prostration. During the study no human endpoints were reached and no animal was sacrificed.

### Animals and housing conditions

To avoid confounding effects associated with urban areas, the study was conducted on captive animals. Eight adult (37.5±2.1 months) male grey mouse lemurs (*Microcebus murinus*) were studied. Animals were born in the laboratory breeding-colony of the National Museum of Natural History in Brunoy, France (48°41′52″ N, 2°30′16″ E) from a stock originally caught in southern Madagascar 45 years ago. Animals were exposed to a natural photoperiodic regimen to entrain the seasonal variations of behaviors, daily rhythms and physiological functions, and were studied during the short-days season, from 8^th^ January to 4^th^ February 2012 (mean civil dawn: 7:56±0:02 AM Gmt+1, mean civil dusk: 6:06±0:03 PM Gmt+1 [35]; civil twilight corresponds to a sun 6° below horizon). They were housed individually in cages (50×30×30 cm), with branches and a nest box, to minimize social influences on biological rhythms. General conditions of captivity were maintained constant: ambient temperature (16–18°C), relative humidity (55%), food in excess including a homemade milky mixture (46 kJ/day) and fresh fruits (29,5 kJ/day) delivered every day during the diurnal resting phase and water available *ad libitum*.

### Light treatments

**Figure 1:**
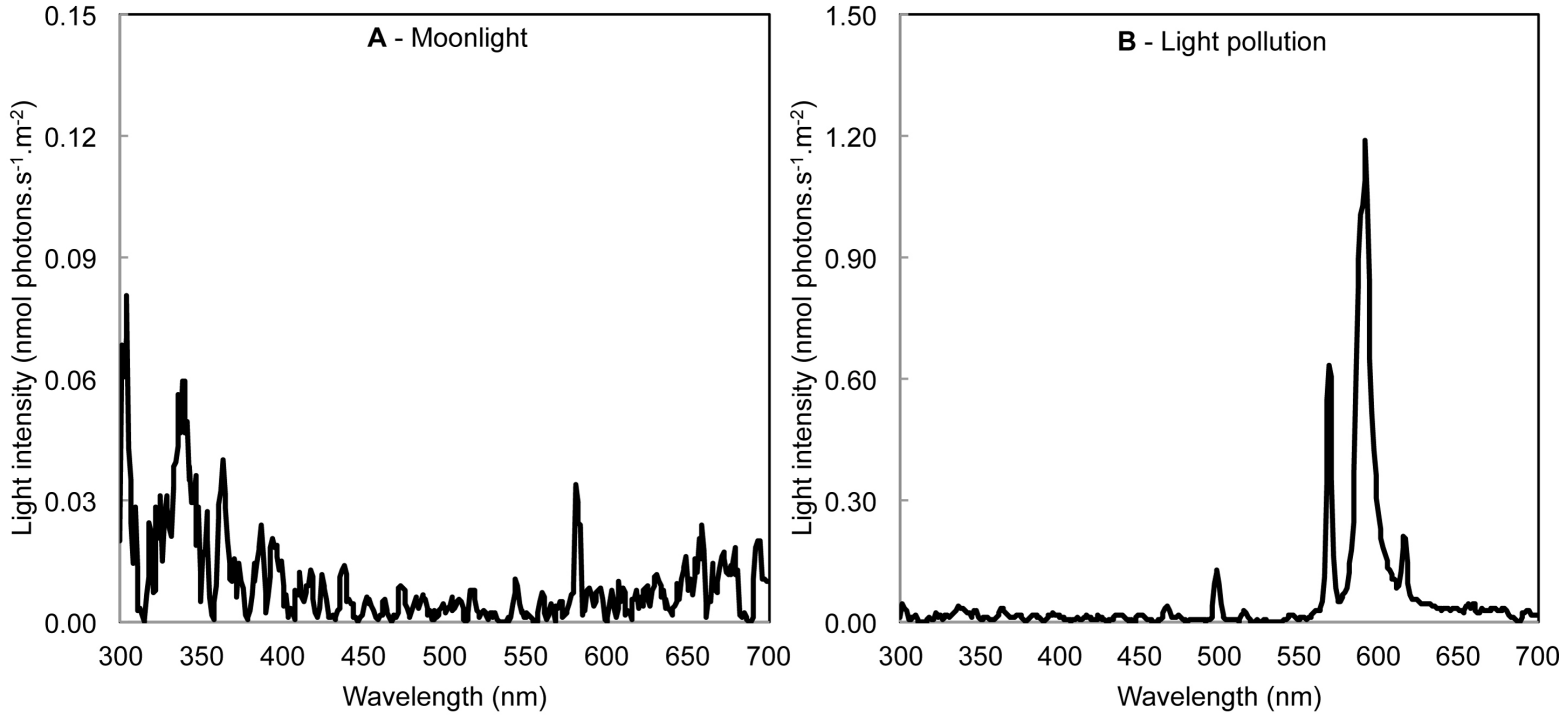
Differences in irradiance spectra of the lights used. To test the impact of urban light pollution on *Microcebus murinus*, we exposed animals to (A) moonlight treatment (light intensity: 3.9±0.1 nmol photons.s^−1^.m^−2^); then to (B) light pollution treatment (light intensity: 24.2±0.9 nmol photons.s^−1^.m^−2^; light of a high pressure sodium streetlight located 50 m in front of the experimental room; peak at 592 nm).

Individual cages were positioned in an experimental room with large bay windows in front of, or away from, streetlights. Due to limited sample size (N = 8), animals were treated as their own control. In order to minimize the impact of external factors during the experiment, all efforts were realized to maintain constant the conditions of captivity. Animals were first exposed 14 nights to the moonlight treatment (MOON) (light intensity: 3.9±0.1 nmol photons.s^−1^.m^−2^ (Figure 1A); the moonlight treatment was representative of the night sky in Brunoy, with a light intensity similar to the full moon (3.5±0.1 nmol photons.s^−1^.m^−2^ [36]). Thereafter, the same animals were exposed 14 nights to the light pollution treatment (POLL) (light intensity: 24.2±0.9 nmol photons.s^−1^.m^−2^ (Figure 1B); the light pollution treatment corresponded to a streetlight located 50 m in front of the cages and positioned 8 m above the ground; spectrum type: high pressure sodium lamp, the most common artificial light used for outdoor lighting characterized by emission lines from 569 to 616 nm [37]; mean timetable switch-on: 5:32±0:03 PM Gmt+1; switch-off: 8:32±0:02 AM Gmt+1). The average light intensity and irradiance spectra were measured at night before and during the experiment using a JAZ spectrometer (Ocean Optics, Dunedin, Florida, USA) between 300–700 nm in triplicate for each individual cage. The spectrometer was placed at the center of the cage in vertical position. During daytime animals were exposed to natural daylight.

### Core temperature and locomotor activity recording

Throughout the study, core temperature (Tc) and locomotor activity (LA) were measured using TA10TA-F20 telemetric transmitters (Data Science Co. Ltd, Saint-Paul, Minnesota, USA) implanted under general anaesthesia (Valium: 1 mg/100 g – sub-cutaneous injection; Ketamine Imalgen: 10 mg/100 g – intra-peritoneal injection; post-operative analgesia, Meloxicam Metacam: 0.02 mg/100 g – sub-cutaneous injection) in the visceral cavity and under veterinary supervision. Calibration for each transmitter was provided by the manufacturer. Experiments were performed after at least two weeks of recovery. A receiving plate (RPC-1, Data Science Co. Ltd, Saint-Paul, Minnesota, USA) positioned in the cage allowed recording of data sent by the transmitter. Tc (in °C) was recorded every 10 min and LA (in arbitrary units) was continuously recorded and summed within this interval by antennas located in the receiving plate and detecting vertical and horizontal movements (X-Y coordinate system, Dataquest Lab Pro v 3.1, Data Science Co. Ltd, Saint-Paul, Minnesota, USA).

**Figure 2:**
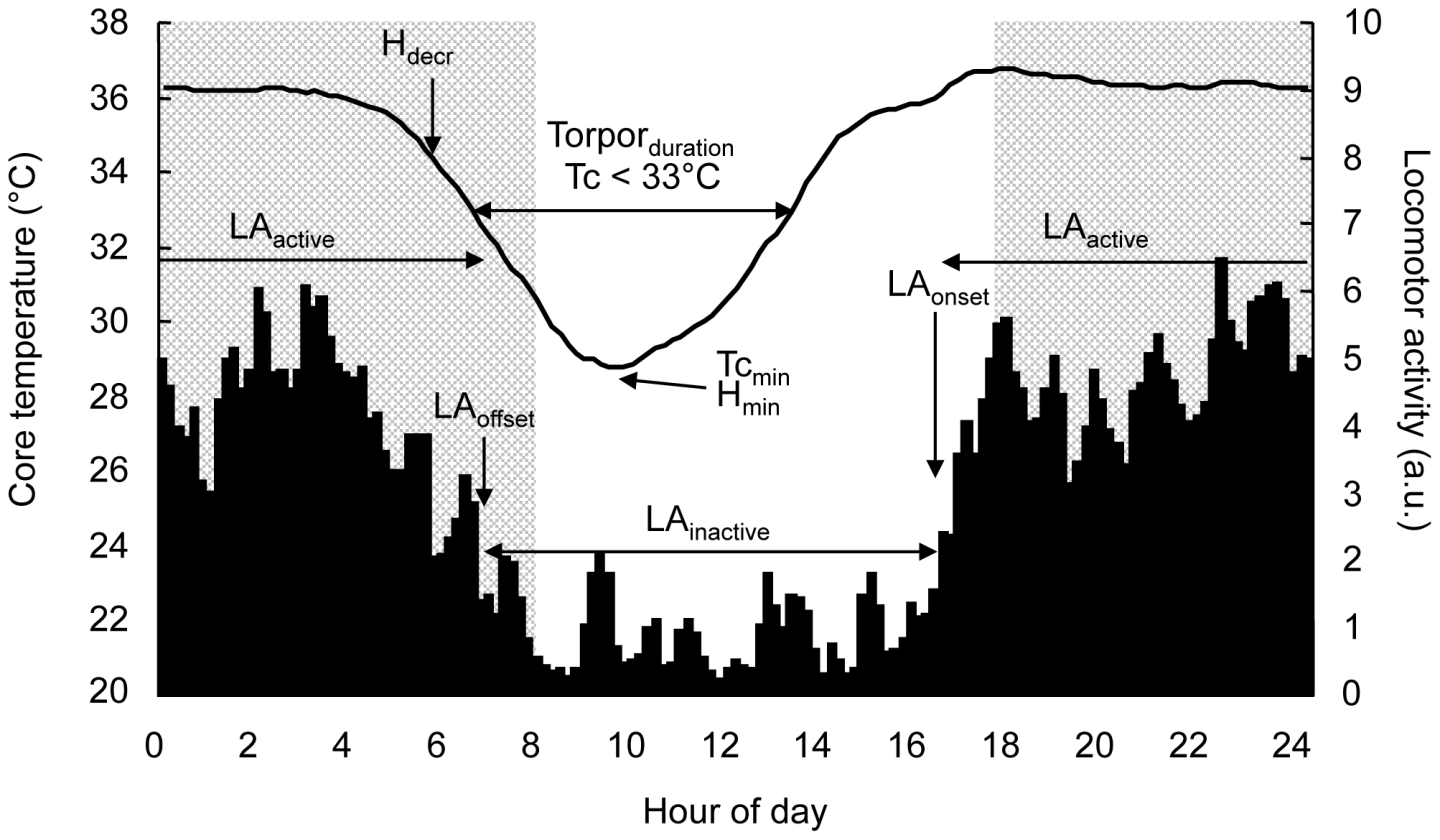
Parameters related to daily rhythms in *Microcebus murinus*. Parameters used to characterize daily rhythms of locomotor activity (LA – arbitrary units) and core temperature (Tc) in mouse lemurs during short-days photoperiod. The night period is indicated by the grey area.

The following parameters were computed for each experimental treatment (MOON and POLL) and each animal, day after day, using graphic determination (Figure 2): in terms of daily rhythm of Tc, we calculated mean Tc during the active nocturnal phase (Tc_active_ in °C), mean Tc during the inactive diurnal phase (Tc_inactive_ in °C), minimal Tc value (Tc_min_ in °C), time of occurrence of Tc_min_ (H_min_ in min), time of onset of Tc drop (H_decr_ in min), duration of the torpor bout (Torpor_duration_ in min) and frequency of the torpor bout (Torpor_frequency_). To determine Tc_active_ and Tc_inactive_, Tc values were averaged during the nocturnal active phase and the diurnal resting phase, respectively between the LA onset and offset and between the LA offset and onset. H_min_ and H_decr_ were expressed in minutes relative to civil dawn. H_decr_ was determined as the first time point after which at least three successive bins of Tc decrease occurred. H_min_ was determined as the time point occurring at least after 30 min of decrease and preceding at least 30 min of Tc increase. Consequently, Tc_min_ corresponded to the Tc value pointed at the H_min_ time point [38]. Torpor_duration_ was computed as the duration with Tc below 33°C (shallow torpor [39]). Torpor_frequency_ corresponded to the number of nights with torpor bouts for each treatment. In terms of daily rhythm of LA, LA onset and offset (LA_onset_, LA_offset_ in min) were defined respectively as the time of occurrence of the first or last three successive bins when activity was greater or lower than LA averaged on 24 hours. LA_onset_ and LA_offset_ were respectively expressed in minutes relative to civil dusk and civil dawn. The duration of the LA nocturnal active phase (α in min) corresponded to the time duration between LA_onset_ and LA_offset_. To determine LA nocturnal intensity and LA diurnal intensity (LA_active_ and LA_inactive_ in arbitrary units), LA values were averaged during the nocturnal active phase and the diurnal resting phase, respectively between LA_onset_ and LA_offset_ and between LA_offset_ and LA_onset_ [38]. For all temporal parameters (H_min_, H_decr_, LA_onset_ and LA_offset_), phase advances and phase delays were respectively expressed by positive or negative values in reference to civil twilight. Civil twilight timing was chosen as reference point because it corresponds to the most rapid changes in light intensity during the daily cycle [40].

### Nocturnal and feeding behaviors recording

For each experimental treatment (MOON and POLL) and each animal, the 14^th^ night, representative of the end of treatment, was filmed using an infrared camera (Handycam HDR-SR7, Sony, Tokyo, Japan) from 4:00 PM, before the animal emerged from its nest box, until at least 30 min after its last return inside, usually around 6:00 AM. To avoid disturbing animals, the camera was positioned during the diurnal resting phase and at one meter from the cage. After recording, the following parameters were computed: in terms of nocturnal behavior, we calculated time of occurrence of the first emergence from the nest box and time of occurrence of the last return inside (Emerge_onset_ and Emerge_offset_ in min) respectively expressed relative to civil dusk and civil dawn and time spent outside the nest box (Time_outside_ in min) during the nocturnal active phase. Animals were considered outside the nest box when their four legs were simultaneously visible. In terms of feeding behavior, time of occurrence of the first feeding and the last feeding bout (Feed_onset_ and Feed_offset_ in min) respectively expressed relative to civil dusk and civil dawn, number of feeding bouts outside the nest box (Feed_n_), time spent feeding outside the nest box (Feed_outside_ in min) and number of fresh fruits slices brought back to the nest box (Fruits_nest_) were determined. For all temporal parameters (Emerge_onset_, Emerge_offset_, Feed_onset_ and Feed_offset_) phase advances and phase delays were respectively expressed by positive or negative values in reference to civil twilight.

### Body mass and caloric intake

Before the experiment, values of body mass were controlled to ensure of their homogeneity (106.9±4.6 g). During each experimental treatment (MOON and POLL), body mass was measured at days 1, 8 and 15 and the body mass slope (Bm_slope_) was computed between days 1 and 15. Daily caloric food intake (CI in kJ) was calculated for each animal from the difference between provided and remaining food mass and was corrected for dehydration. Bm_slope_ was representative of changes in body condition and CI was representative of feeding behavior.

### Statistical analysis

Because we used temporal repeated measures on the same animals, Tc_active_, Tc_inactive_, Tc_min_, H_min_, H_decr_, Torpor_duration,_ LA_onset_, LA_offset_, α, LA_active_, LA_inactive_ and CI parameters were analyzed using linear mixed effects models, built with the ‘lme’ function (package ‘nlme’) [41]. To explore the effect of light pollution on these parameters throughout the exposure and to determine if this effect could vary over time, the starting model including the effect of light pollution, the effect of time (in numbers of day since the beginning of the experiment) and the effect of the interaction between light pollution and time has been constructed. To take into account inter-individual variability, the effect of individual identity was declared as a random effect. To take into account temporal autocorrelation among model's residuals, an auto-regressive structure of order 1 (‘corAR1’ function) was included into the model. The final model, containing only significant effect(s), was obtained by deletion of the non-significant effect(s) from the starting model. Normality of final model's residuals was checked with a normal quantile-quantile plot and homoscedasticity with boxplot of parameters' residuals according to treatment and boxplot of parameters' residuals according to days [41]. Other parameters including Torpor_frequency_, Emerge_onset_, Emerge_offset_, Time_outside_, Feed_onset_, Feed_offset_, Feed_n_, Feed_outside_, Fruits_nest_ and Bm_slope_ were analyzed using, when appropriate, Student paired *t*-test or Wilcoxon paired test. All these parameters were checked for normality with the Shapiro-Wilk test. The probability level for statistical significance was *P*<0.05. All analyses were performed with R version 2.14.2 (R Development Core Team, 2001). Results are presented as means ± standard error of the means (Table 1).

**Table 1:** Means ± SEM of parameters and related statistics.

Category	Parameters	Moonlight	Light pollution	L-ratio	*β*	*P*
Core temperature	Tc_active_, °C	36.3±0.04	36.6±0.04	14.2	0.3±0.07	<0.0001
	Tc_inactive_, °C	32.3±0.4	34.6±0.2	13.4	2.2±0.5	<0.0001
	Tc_min_, °C	27.5±0.7	32.2±0.4	20.5	4.6±0.9	<0.0001
	H_min_, min	−130.3±10.7	−166.3±12.7	3.9	−36.2±16.5	<0.05
	H_decr_, min	139.3±10.1	57.7±6.1	25.1	−82.2±14.9	<0.0001
	Torpor_duration_, min	201.4±21	70.1±13.4	15.9	−134.1±32.4	<0.0001
	Torpor_frequency_, a.u.	7.6±1.4	3.9±0.9			<0.05
Locomotor activity	LA_onset_, min	88±3.2	32.1±3.6	48.4	−55.0±5.8	<0.0001
	LA_offset_, min	125.5±7.5	60.3±5.6	22.2	−63.3±12.1	<0.0001
	α, min	794.9±8	798.2±7.1	0.02	1.7±12.4	0.88
	LA_active_, a.u.	4.8±0.3	3.4±0.1	8.5	−1.3±0.3	<0.0001
	LA_inactive_, a.u.	0.8±0.1	0.6±0.1	0.9	−0.2±0.2	0.34
Nocturnal behavior	Emerge_onset_, min	97.4±7.9	21.6±4.2			<0.01
	Emerge_offset_, min	203.7±7.9	158.4±7.1			<0.01
	Time_outside_, min	464.4±67.3	226.3±53.8			<0.05
Feeding behavior	Feed_onset_, min	82.1±9.8	13.7±7.7			<0.01
	Feed_offset_, min	293.6±29.7	232.1±14.9			<0.05
	Feed_n_, a.u.	18.7±4	19.1±1.6			0.67
	Feed_outside_, min	19.7±4.8	13.4±1.9			0.29
	Fruits_nest_, a.u.	1.4±0.3	2±0.3			0.22
	CI, kJ	74.5±0.3	71.8±0.7	0.6	−0.4±0.5	0.45
Body condition	Bm_slope_, a.u.	−0.7±0.1	−0.8±0.1			0.68

Means ± SEM of parameters (daily rhythms, behaviors and body condition) in animals during moonlight treatment and then exposed to light pollution. Statistics related to linear mixed effects models are indicated. Significant differences are indicated in bold.

## Results

### Effects of light pollution on daily rhythms: core temperature

**Figure 3:**
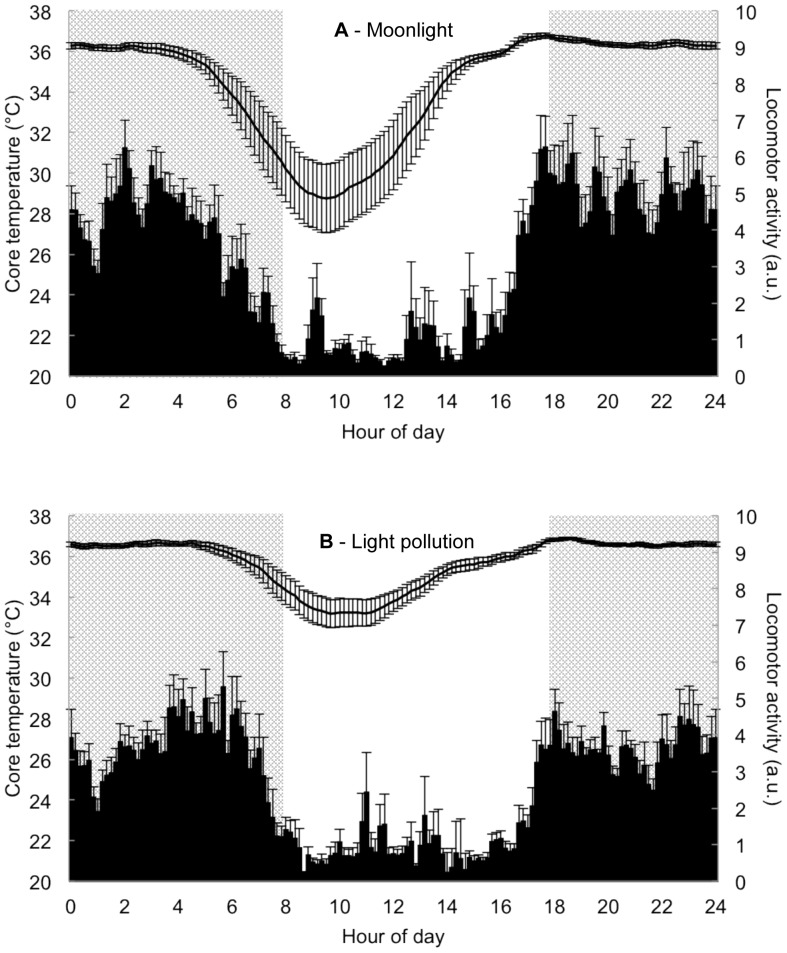
Means ± SEM of daily rhythms in mouse lemurs exposed to moonlight *versus* light pollution treatment. Mouse lemurs (N = 8) exposed to moonlight treatment (A) and then to light pollution treatment (B) at night (grey areas) during short-days photoperiod. Exposure to light pollution significantly delays the onset/offset of locomotor activity, decreases the nocturnal intensity of locomotor activity and increases both diurnal and nocturnal core temperatures.

In terms of daily rhythm of core temperature, all parameters were affected by light pollution throughout the two weeks of exposure without direct effect or interaction with time. In POLL, the mean Tc during the active nocturnal phase, the mean Tc during the inactive diurnal phase and the minimal Tc value were significantly higher than in MOON (respectively 0.3°C, 2.2°C and 4.5°C higher on average in POLL). In POLL, the time of onset of Tc drop and the time of occurrence of Tc_min_ were reached significantly later than in MOON (respectively 79.7 min and 36.2 min later on average in POLL). These results indicate that H_decr_ and H_min_ were delayed in POLL. In addition, in POLL the duration of the torpor bout and the frequency of the torpor bout were significantly lower compared to MOON (respectively 130.5 min lower and 2 times less frequent on average in POLL) (Table 1; Figure 3A,B).

### Effects of light pollution on daily rhythms: locomotor activity

All parameters of the daily rhythm of locomotor activity, except the duration of the LA nocturnal active phase and the LA diurnal intensity, were affected by light pollution throughout the two weeks of exposure. For all parameters there was no direct effect or interaction with time. In POLL, the LA onset and the LA offset began and stopped respectively and significantly later than in MOON (respectively 56.3 min and 63.7 min later on average in POLL). However, there was no significant difference for the duration of the LA nocturnal active phase. In POLL, the LA nocturnal intensity was significantly lower compared to MOON (1.4 times lower on average in POLL). However, there was no significant difference for the LA diurnal intensity. These results indicate that light pollution had a negative effect on LA intensity at night, but not during the day (Table 1; Figure 3A,B).

### Effects of light pollution on nocturnal behavior

All parameters of nocturnal behavior were affected by light pollution after two weeks of exposure. In POLL, the time of occurrence of the first emergence from the nest box and the time of occurrence of the last return inside the nest box occurred significantly later compared to MOON (respectively 75.8 min and 45.4 min later on average in POLL). In POLL, the time spent outside the nest box was significantly shorter than in MOON (2 times shorter on average in POLL). These results indicate a phase delay for Emerge_onset_ and Emerge_offset_ in POLL and that light pollution had a negative effect at night on time spent outside the nest box (Table 1).

### Effects of light pollution on feeding behavior and body mass

For feeding behavior, the time of occurrence of the first feeding and the last feeding bout were affected by light pollution after two weeks of exposure, but not the other parameters. In POLL, the time of occurrence of the first feeding bout and the time of occurrence of the last feeding bout occurred significantly later compared to MOON (respectively 68.4 min and 61.5 min later on average in POLL). These results indicate that Feed_onset_ and Feed_offset_ were delayed in POLL. There was no significant difference in number of feeding bouts outside the nest box, time spent feeding outside the nest box, number of fresh fruits slices brought back to the nest box and in daily caloric food intake between MOON and POLL. Finally, light pollution had no effect on body mass. There was no difference in the body mass slope between MOON and POLL (Table 1).

## Discussion

### Effects of light pollution on daily rhythms: core temperature

For the first time it was demonstrated in a nocturnal primate that light pollution modifies the daily rhythm of core temperature. Core temperatures, both during the night and during the daily rest, were significantly higher under exposure to light pollution. In addition, the daily phase of hypothermia (torpor) was delayed and less pronounced corresponding to a long-days phenotype, *i.e.* a summer phenotype, suggesting an alteration of seasonal acclimatization. Indeed, in mouse lemurs, the patterns of core temperature and daily torpor are photoperiod-dependent with higher frequency and duration of torpor in short-days photoperiod compared to long-days photoperiod [34]. Furthermore, it is important to note that the increase in core temperature is not related to an exercise-associated thermogenesis or diet-induced thermogenesis since mouse lemurs exposed to light pollution were less active and did not eat more than during the moonlight treatment (see discussion below). These results suggest an alteration of thermoregulation related to exposure to light pollution. Similar results were obtained in the social vole (*Microtus socialis*), indicating that nocturnal light pulses may act as a stressor imposing a threat to the physiological homeostasis and, especially, negatively affect winter acclimatization of thermoregulatory mechanisms probably by mimicking summer acclimatization [42], [43]. The authors named this phenomenon ‘seasons out of time’ [43]. In mouse lemurs, thermoregulation, especially daily torpor, is an important and key mechanism to cope with adverse ambient temperature and low food availability [38], [39], [44], [45]. Consequently, increased core temperature and reduced torpor bouts under light pollution could lead to an increase in energy expenditures and, thus, could minimize successful adaptation to seasonal environmental changes [18], [19]. However, a complementary study is needed to strongly support this phenomenon of ‘seasons out of time’ in mouse lemurs. The study of seasonal photoperiodic responses as sexual activity will be very informative to illuminate this hypothesis. In this sense, we conducted a preliminary study on the impact of light pollution on reproductive parameters in both sexes (testosterone, oestradiol, oestrus cycle) in mouse lemurs. Results (unpublished data) indicated significant advances in seasonal activation of reproductive function in animals exposed to light pollution compared to control animals. These complementary results suggest that light pollution could desynchronize seasonal processes and alter seasonal acclimatization in mouse lemurs.

### Effects of light pollution on daily rhythms: locomotor activity

Light pollution significantly modifies the daily rhythm of locomotor activity. Although its total duration was not modified, locomotor activity presented a delay in both its onset/offset and was significantly reduced at night under exposure to light pollution. Such changes were observed in bats [23]–[25] and in nocturnal rodents exposed to artificial light at night [22], [26], [30], [46]–[48] leading to modification of habitat use and inter/intraspecific competition [5], [6], [26]. These results could illustrate a desynchronization of the activity patterns with the geophysical cycles of the environment or a negative light masking effect as described by Rotics *et al* [31]. Indeed, light stimulus can override an animal's internal biological clock and consequently modify the activity patterns. In nocturnal species, light stimulus suppresses activity [49]. Furthermore, light pollution could be problematic considering activities related to moonlight. Recent studies have demonstrated in several taxa, including mammals, that moonlight and lunar cycle have an effect on activity rhythm, synchronization of reproduction, communication, navigation, habitat use, foraging and predation [17], [50]–[52]. Accordingly, beyond altering the natural light/dark and its perception by living organisms, light pollution could also affect the perception of moonlight and lunar cycle. Rich and Longcore evoke this phenomenon as a ‘perpetual full moon’ [5] and Cinzano *et al.* denounce a ‘perennial artificial moonlight’ caused by light pollution [53].

### Effects of light pollution on nocturnal behavior

Changes in patterns of locomotor activity were associated with modifications in nocturnal behaviors of mouse lemurs exposed to light pollution which could suggest a behavioral inhibition. The time spent outside the nest box was significantly lower and the occurrences of the first emergence from the nest box and the last return inside were significantly delayed with light pollution. For the occurrence of the first emergence from the nest box, video recordings showed that animals exposed to light pollution spent several minutes at the entrance before emerging. This behavior has been observed in the flying squirrel (*Glaucomys volans*) whose emergence when exposed to artificial light was delayed by 40 minutes [47]. As in most small nocturnal mammals [17], light pollution induces a repulsive response in *M. murinus*. Under natural conditions, such behavior would be related to an anti-predator behavior. Indeed, light affects visual abilities of nocturnal predators and preys and the perceived risk of predation increases with illumination of the environment [54], [55]. Consequently, decreases in locomotor activities reduce the animal's ability and willingness to exploit its environment. Moreover, light pollution contributing to fragment the environment would modify dispersal in nocturnal mammals as shown in the Puma (*Puma concolor*) that move away from the urban artificial light to the darkest areas [56].

### Effects of light pollution on feeding behavior and body mass

Light pollution partially alters feeding behavior in mouse lemurs. Animals fed significantly later, tended to spend less time feeding outside and brought more fruits in the nest box under light pollution. These changes did not result in a significant impact on the daily caloric food intake or on body mass. However, in the wild, where food availability is uncertain, it has been demonstrated in bats and nocturnal rodents that light pollution significantly reduces feeding behavior and body mass. In the Darwin's leaf-eared mouse (*Phyllotis darwini*), animals exposed to simulated moonlight carried 40% of their food to the refuge site against only 4% in dark conditions [57]. More, animals exposed to simulated moonlight consumed 15% less food during the experiment and lost 4.4 g in body mass in only one trial night against 1.1 g in dark conditions. In bat colonies (*Myotis emarginatus*, *M. oxygnathus*) exposed to light pollution, juvenile growth was slowed in association with reduced body mass [23]. It could be suggested that the shift in time for feeding behavior could lead animals to miss peaks of food availability and to generate interspecific competition. In natural communities, foraging times are partitioned among species that prefer different levels of lighting. But if natural lighting is altered by light pollution, this partitioning is changed and species that were not previously in competition could become competitors [5], [6]. Light pollution could consequently be detrimental to photo-sensitive species [4]. For example, the bat *Pipistrellus pipistrellus*, which tolerates artificial light, is able to benefit from the aggregation of nocturnal insects around streetlights but not the bat *Rhinolophus hipposideros*, which does not tolerate artificial light, generating competitive exclusion for food resources [27]. In an opposite way, Fonken *et al.* demonstrated in male mice that low levels of light at night disrupt the timing of food intake leading to increased weight gain and induce metabolic alterations as impaired glucose tolerance [58]. These results ask questions about the impact of light pollution on animal fitness, notably considering that increased body mass and reduced glucose tolerance are indicative of a prediabetic-like state [59].

## Conclusions

For the first time in a nocturnal primate, urban light pollution was demonstrated to modify the expression of biological rhythms, nocturnal and feeding behaviors and to have a negative impact on thermoregulation and potentially energy balance through changes in patterns of daily torpor bouts. Evidence of desynchronization of both daily and seasonal biological rhythms could have deleterious consequences for animals, especially in terms of adaptation and anticipation of environmental changes. By altering behaviors, light pollution could affect the ability of individuals to effectively exploit their environment and its resources, and contribute to fragment the habitat particularly for photo-sensitive species. Ultimately, survival, reproduction and fitness of these species could be altered. At the ecological scale, the interspecific equilibrium could also be threatened.
